# Cannabidiol-Loaded Retinal Organoid-Derived Extracellular Vesicles Protect Oxidatively Stressed ARPE-19 Cells

**DOI:** 10.3390/biomedicines13051167

**Published:** 2025-05-10

**Authors:** Peggy Arthur, Sangeetha Kandoi, Anil Kalvala, Breana Boirie, Aakash Nathani, Mounika Aare, Santanu Bhattacharya, Tanmay Kulkarni, Li Sun, Deepak A. Lamba, Yan Li, Mandip Singh

**Affiliations:** 1 College of Pharmacy and Pharmaceutical Sciences, Florida A&M University, Tallahassee, FL 32307, USA; peggy1.arthur@famu.edu (P.A.); anil.kalval@famu.edu (A.K.); breana1.boirie@famu.edu (B.B.); aakash1.nathani@famu.edu (A.N.); mounika1.aare@famu.edu (M.A.); 2 Department of Ophthalmology, University of California San Francisco, San Francisco, CA 94143, USA; somprak2@jh.edu (S.K.); lamba.deepak@gene.com (D.A.L.); 3 Eli and Edythe Broad Center of Regeneration Medicine and Stem Cell Research, University of California San Francisco, San Francisco, CA 94143, USA; 4 Solomon H. Snyder Department of Neuroscience, Johns Hopkins University, Baltimore, MD 21218, USA; 5 Department of Biochemistry and Molecular Biology, Mayo College of Medicine and Science, Jacksonville, FL 32224, USA; bhattacharya.santanu@mayo.edu (S.B.); kulkarni.tanmay@mayo.edu (T.K.); 6 Department of Physiology and Biomedical Engineering, Mayo College of Medicine and Science, Jacksonville, FL 32224, USA; 7 Department of Chemical and Biomedical Engineering, FAMU-FSU College of Engineering, Tallahassee, FL 32310, USA; li.sun@med.fsu.edu; 8 Department of Biomedical Sciences, College of Medicine, Florida State University, Tallahassee, FL 32306, USA; 9 Immunology and Regenerative Medicine, Genentech, South San Francisco, CA 94080, USA

**Keywords:** extracellular vesicles, retinal organoids, cannabinoid, small RNA profiling, oxidative stress, ARPE-19 cells, AMPK

## Abstract

**Background/Objectives:** Age-related macular degeneration (AMD) is the third leading cause of irreversible blindness in elderly individuals aged over 50 years old. Oxidative stress plays a crucial role in the etiopathogenesis of multifactorial AMD disease. The phospholipid bilayer EVs derived from the culture-conditioned medium of human induced pluripotent stem cell (hiPSC) differentiated retinal organoids aid in cell-to-cell communication, signaling, and extracellular matrix remodeling. The goal of the current study is to establish and evaluate the encapsulation of a hydrophobic compound, cannabidiol (CBD), into retinal organoid-derived extracellular vesicles (EVs) for potential therapeutic use in AMD. **Methods:** hiPSC-derived retinal organoid EVs were encapsulated with CBD via sonication (CBD-EVs), and structural features were elucidated using atomic force microscopy, nanoparticle tracking analysis, and small/microRNA (miRNA) sequencing. ARPE-19 cells and oxidative-stressed (H_2_O_2_) ARPE-19 cells treated with CBD-EVs were assessed for cytotoxicity, apoptosis (MTT assay), reactive oxygen species (DCFDA), and antioxidant proteins (immunohistochemistry and Western blot). **Results:** Distinct miRNA cargo were identified in early and late retinal organoid-derived EVs, implicating their roles in retinal development, differentiation, and functionality. The therapeutic effects of CBD-loaded EVs on oxidative-stressed ARPE-19 cells showed greater viability, decreased ROS production, downregulated expression of inflammation- and apoptosis-related proteins, and upregulated expression of antioxidants by Western blot and immunocytochemistry. **Conclusions:** miRNAs are both prognostic and predictive biomarkers and can be a target for developing therapy since they regulate RPE physiology and diseases. Our findings indicate that CBD-EVs could potentially alleviate the course of AMD by activating the targeted proteins linked to the adenosine monophosphate kinase (AMPK) pathway. Implicating the use of CBD-EVs represents a novel frontline to promote long-term abstinence from drugs and pharmacotherapy development in treating AMD.

## 1. Introduction

Age-related macular degeneration (AMD) primarily affects the central vision of the human eye, responsible for providing high-acuity and color vision. AMD is considered the third leading cause of irreversible blindness in individuals aged >50 years old [1,2] and is growing rapidly as a global crisis, with ~288 million individuals estimated to be affected by 2040 [3]. AMD has two categories—dry (atrophic) being the most common and wet (neovascular or exudative) being the least common—but both cause severe vision impairment and progressive vision loss [4]. The likelihood of developing AMD is impacted by a combination of factors, including advanced age and inherited traits through the family history [5,6]. Furthermore, modifiable aspects of lifestyle, including smoking [7], poor nutritional intake [8,9], obesity [10], and lack of physical activity [11], increase the risk of AMD, making it a complex interplay with hereditary elements. The two most significant risk factors triggering AMD are oxidative stress [12,13,14] and senescence [15]. A growing body of evidence suggests that inflammation is indispensable in progressing the pathogenesis of AMD, causing degeneration of retinal pigment epithelium (RPE)/photoreceptors [16,17,18]. When the cellular production of reactive oxygen species (ROS) exceeds the antioxidant defenses, the normal function is disrupted, initiating chronic oxidative stress to cellular senescence and senescence-associated secretory phenotype (SASP). A collection of inflammatory molecules released by senescent cells, including growth hormones, cytokines, chemokines, and proteases, contributes to the pathogenesis of AMD [19].

Both the retina and RPE tissues lining the inner region of the posterior ocular segment are persistently exposed to light for visual signal processing, thereby consuming more oxygen for the oxidation of polyunsaturated fatty acids and phagocytosis of photoreceptor’s outer segments by RPE. Under such conditions, the homeostasis of a normal cellular metabolic process is endangered by oxidative stress insults via the accumulation of ROS [20]. Over the last few years, pharmacological and pharmacokinetic investigative studies have confirmed the clinical effectiveness of cannabinoids (CBD) as a safe drug for human use. For instance, CBD has been widely reported as a neuroprotective antioxidant [21,22] to inhibit inflammation [23,24] via the activation of the N-methyl-D-aspartate (NMDA) receptor [25]. A study reported that administration of CBD in collagen-induced arthritis mice could modulate the immune system, decreasing the release of interleukin-1 and TNF-α to exert anti-inflammatory effects [24]. Another study’s findings showed that the CBD-treated ischemic gerbils promoted the survival of the central nervous system’s CA1 neurons, thereby presenting CBD as an ‘anti-ischemic drug’ [26]. Moreover, intravenous injections of CBD in rats with NMDA-induced retinal neurotoxicity attenuated the peroxynitrite formation and prevented the NMDA-induced apoptosis in the retina [27]. In a study conducted by El-Remessy et al. (2006) [28], CBD treatment reversed the retinal inflammation and oxidative stress in a streptozotocin-induced diabetic rat model, which displayed pathologies of increased oxidative stress, retinal cell death, and vascular hyperpermeability concomitant with higher levels of VEGF, ICAM-1, and TNF-α, along with the activation of the p38 MAP kinase pathway [28].

Extracellular vesicles (EVs), including exosomes, microvesicles, and apoptotic bodies, are a group of lipid bilayer macromolecules released by cells. EV’s size (30–1000 nm), biogenesis, and their putative roles vary on the cells’ origin, source, and age [29,30]. EV cargo composition is complex and enriched with proteins, nucleic acids including DNA, RNA, microRNA (miRNA), fragmented mRNA, small non-coding RNAs, long non-coding RNA, small nucleolar RNA, Y RNA, mitochondrial RNA, vault RNA, and unique lipids [31]. EVs are essential for intercellular communication as they transport the genetic and proteomic information between cells and organs, regulating the signaling pathways [32]. Due to numerous complementary characteristics, EVs have emerged as a promising therapeutic tool for treating various neurodegenerative diseases. Some of them include (i) low immunogenicity profile, (ii) the ability to pass through the blood–brain barrier, (iii) stability in circulating biological fluids due to their widespread distribution, (iv) a unique surface chemistry enabling the targeting capabilities, (v) exerting functional responses by transferring the cargo across target cell membranes, and (vi) mediating the intercellular transfer of mRNAs and miRNAs [28,31,33]. Despite several known studies demonstrating the therapeutic benefits of EVs, very little is known about drug-loaded EVs for treating ocular diseases.

EVs and liposomes possess similar structural features with lipid bilayer composition for encapsulating the therapeutic agents (hydrophobic and hydrophilic molecules) within their membrane structures to effectively deliver drugs [29]. Nevertheless, in contrast to the liposome system, EVs exhibit low immunogenicity, minimum cytotoxicity, and a lack of mutagenicity issues, making them a superior system for drug delivery [30]. Lastly, unlike the requirement of nano formulations for liposomes and dendrimers, EVs are released in vitro by 2D or 3D culture systems and can be a valuable source to generate a specialized drug delivery system. Cell culture-derived EVs can be loaded with a drug of choice at ease for medicating small molecules due to their small size, lower toxicity, and biocompatibility. Compared to administering drugs per se, EVs encapsulated with targeted drugs offer an innovative system to improve the pharmacokinetic and pharmacodynamic properties, particularly in anticancer treatments [32,34,35,36]. Our previous study demonstrated the beneficial pharmacological effects of CBD and CBD-loaded human umbilical cord mesenchymal stem cell (hUCMSC)-EVs on paclitaxel (PTX)-treated mice displaying pathophysiological indices of neuropathy [37]. Treatment of PTX-treated mice with CBD and CBD-EVs enhanced the antioxidant expression and lowered the NF-kB phosphorylation in the dorsal root ganglion cells (DRG) and spinal tissue homogenates [37].

Our earlier work laid down the pipeline for optimizing the preparation of EVs from human-induced pluripotent stem cell (hiPSC)-derived 3D retinal organoid systems along with cataloging differentially expressed protein profiles on EVs [34]. The current study investigates the impact of CBD-encapsulated retinal organoid EV applications on hydrogen peroxide (H_2_O_2_)-stressed ARPE-19 cells in regulating inflammation at the cellular and molecular levels. To the best of our knowledge, this is the first report establishing the counteracting antioxidative signaling from CBD-encapsulated retinal organoid EVs in an oxidatively stressed 2D culture model. The findings from this study have expanded and supported the clinical utility of CBD and CBD-EVs as potential therapeutic agents in the treatment of AMD.

## 2. Materials and Methods

**Figure 1:**
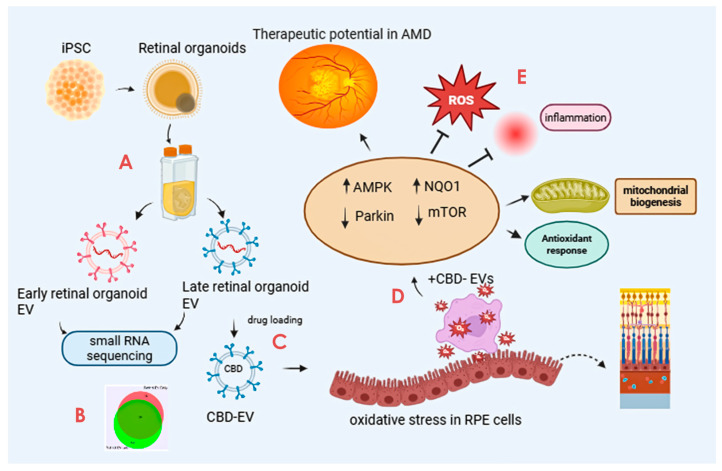
Graphical illustration of the study. The study highlights the promising avenue of CBD-EVs and their potential future perspective for therapeutic use in treating AMD. The key step includes (A) isolation of EVs from iPSC-derived retinal organoids CCM cultured in a PBS-vertical wheel bioreactor. (B) Characterization of isolated EVs by small RNA (miRNA) sequencing. (C) Formulation of CBD encapsulation within the EVs. (D) Assessing the anti-apoptotic and antioxidant effects of CBD, CBD-EVs, and EVs in oxidative-damaged ARPE-19 cells. (E) Cell lysates of CBD- and CBD-EV-treated oxidative-damaged ARPE-19 cells demonstrating increased expression of SOD2, NQO1, and HO1 to regulate mitochondrial biogenesis and attenuate oxidative stress and inflammation via AMPK-activated NRF2 and NF-κB signaling pathways. This figure was created in Biorender.

The scope and illustration of the study are shown in Figure 1.

### 2.1. Isolation of EVs from Retinal Organoids

EVs were extracted from culture-conditioned medium (CCM) of 3D-retinal organoids (early and late) differentiated from hiPSCs by a modified differential centrifugation method using polyethylene glycol (PEG) precipitation, as shown in our previously published and other studies [34,38,39]. To eliminate the bigger contaminants, the CCM was differentially centrifuged at 500× *g* for 5 min, 2000× *g* for 10 min, and 10,000× *g* for 30 min, and the pellet was discarded. After the final centrifugation, the supernatant was incubated with equal volumes of PEG 6000 solution (16% wt/vol PEG, Thermo Scientific, Waltham, MA, USA; A17541.01 and 1M NaCl, Sigma-Aldrich, St. Louis, MO, USA; S3014) overnight at room temperature to concentrate and produce small EVs. The CCM and PEG solutions were centrifuged at 3214× *g* for one hour to extract EVs. EV pellets were resuspended in 1X phosphate-buffered saline (PBS) and stored at 4 °C for immediate use or −80 °C for long-term storage. Based on the birth and development of retinal cell types from our previous staging studies, organoids utilized for this study were classified as early-stage (D-Days; D50-D90) and late-stage (>D90) [34].

### 2.2. Small RNA Profiling of EVs

EV samples (from early and late retinal organoids) were treated with RNase (ThermoFisher, Waltham, MA, USA); AM2294) at 50 ng/mL final concentration for 30 min at room temperature. After the treatment, RNase inhibitor ( New England Biolabs, Ipswich, MA, USA; M0314) and PCR-grade water were added to make up the total volume to 200 μL. According to the manufacturer’s instruction, miRs were isolated by adding 600 μL Trizol LS (ThermoFisher; 10296010). To effectively increase the yield of small RNAs, three volumes of 100% ethanol and linear acrylamide (VWR, Radnor, PA, USA; 97063–560) were utilized instead of isopropyl alcohol and incubated overnight at −20 °C. The isolated RNAs were then quantified using a Qubit microRNA assay kit (ThermoFisher; Q32880). Small RNA libraries were generated using the NEBNext^®^ Multiplex Small RNA library prep set from Illumina (NEB; E7300). To effectively increase the yield and prevent the primer/adaptor dimer, the 3′ SR primer was diluted to 1:5 and ligation time was extended overnight at 16 °C. The HS DNA chip and KAPA library quantification kit were used before illumina NovaSeq 6000 sequencing at the Florida State University College of Medicine Translational Laboratory. Each sample was analyzed in triplicate.

### 2.3. RNA-Seq Data Analysis

Raw data of miR-seq were uploaded on OASIS, a web-based online miR analysis tool, to identify and map the small RNAs on the human reference genome hg38. Differentially expressed miRs were analyzed by both OASIS and miRNet using default settings. RNA-seq data were processed and analyzed using NetworkAnalyst software v3.0 for comprehensive gene expression profiling and analysis. After filtering the unannotated genes and annotated genes with <10% variance and <10 counts, the remaining genes were normalized against log2 counts per million. Differentially expressed genes (DEGs) were identified using the DESeq2 software package (1.24.0) to identify significantly expressed genes between different groups of samples. Heatmaps of global DEGs and gene-enriched pathways were also visualized.

### 2.4. Preparation of CBD Encapsulation into EVs

Similarly to our earlier study, an optimized sonication technique was applied to encapsulate the CBD on the late retinal organoid EVs (CBD-EVs) [30,37]. Late retinal organoid EVs were used for CBD encapsulation due to the higher expression of exosome biogenesis genes and markers based on our previous study [34]. Blank EVs were stabilized in 0.1% BSA (Sigma, A5611) [made in 10% sucrose solution (*w*/*v*)] to produce a stable formulation without precipitation on an ice bath. Blank EVs (1.5 × 10^11^ particles/mL) were then dispersed in ethanolic CBD (Purisys^TM^, Athens, GA, USA; NQS1951) solution (10% *v*/*v* protein content) and subjected to sonication. The sonication condition included 20% amplitude, 3 cycles of 30 s on/off for a total of 2 min with 5 min intermittent cooling on an ice bath between each cycle, followed by the incubation at 22 °C for 1 h [30,37]. CBD-EVs were filtered through a sterile 0.22 µm membrane filter for further studies.

### 2.5. Characterization of CBD-Loaded EVs by Nanoparticle Tracking Analysis

The physical characteristics, including the size, number of particles, and zeta potential of CBD-EVs, were assessed using nanoparticle tracking analysis (NTA) and Zeta View equipment (Zeta View^®^ TWIN PMX-220, Ammersee, Germany). Evaluation of mean particle size and zeta potential was assessed by utilizing a dynamic light scattering approach at 25 °C and a 90° angle. Zeta view analysis was used to process the images and videos. All the formulations were diluted in particle-free 1X PBS at 1:1000 and examined in triplicate.

### 2.6. Drug Loading and Entrapment Efficiency of CBD-EVs

An ultracentrifugation approach was used to separate unbound CBD (free drug) from encapsulated CBD prior to assessing the entrapment efficiency of CBD-EVs. Briefly, the CBD-EV formulation was centrifuged at 120,000× *g* for 1 h. The supernatant was collected and analyzed as an unbound CBD drug. The entrapped drug released from the EVs was dissolved in lysis buffer (RIPA buffer with 1% protease and phosphatase) and sonicated using a probe sonicator for 1 min. The concentration of unbound CBD in the formulation was assessed using reversed-phase high-performance liquid chromatography (RP-HPLC; Waters, Milford, MA USA) [30]. Different concentrations of CBD standards (0.5, 1, 2, 4, 8, 16, and 32 µg/mL) were prepared from CBD stock solution (1 mg/mL diluted in methanol). Each sample containing 20 µL of CBD was captured on a Symmetry^®^ C18 column (150 3.9 mm, 5 µm; Waters Corporation, Milford, MA, USA) after injection. In an isocratic mode, a flow rate of 1 mL/min was employed with the mobile phase of methanol/water (85:15; *v*/*v*). Using a photodiode array (PDA) detector, the concentration was estimated at a 220 nm wavelength [30].

### 2.7. Atomic Force Microscopy

As per our previous studies, EV samples were prepared for atomic force microscopy (AFM) [34,37]. We employed peak-force quantitative nanomechanical mapping (PFQNM) to study the behavior of CBD-EVs in a fluid environment and assessed the morphological attributes, including the lateral and longitudinal measurements, the 3D height profile, and surface roughness. Surface topography was acquired using a ScanAsyst Air purchased from Bruker ( Santa Barbara, CA, USA), a sharp pyramidal geometry probe bearing a nominal tip of 5 nm radius. Experimental parameters involving a scan rate of 0.1 Hz and a peak force setpoint of 300 pN were applied for morphological assessment. Post-acquisition of topography images, Nanoscope v1.9 software was utilized to analyze the images (N = 10).

### 2.8. ARPE-19 Cell Culture, Viability, and Cytotoxicity Assay

ARPE-19 cells (ATCC, Manassas, VA, USA, CRL-2302) were cultured in 75 cm^2^ flasks with DMEM/F12 (Genessee Scientific, El Cajon, CA, USA; 25-503), 10% FBS (ATCC, 30-2020), and 100 U/mL penicillin–streptomycin (Sigma-Aldrich, St. Louis, MO, USA; P4333) at 37 °C, 5% CO_2_, and 95% humidity. The culture medium was changed every other day. Upon 80% confluency, the cells were passaged using Trypsin-EDTA (Sigma, T4049) and cultured in 96-well plates (8 × 10^3^ cells/well). After 24 h of plating, the ARPE-19 cells were incubated in culture medium with differing concentrations (100, 200, 300, 400, and 500 µM) of H_2_O_2_ (Sigma, H1009) for 24 and 48 h at 37 °C, 5% CO_2_, and 95% humidity. Cells not treated with H_2_O_2_ were used as a control. Following 24 and 48 h, the viability of ARPE-19 cells was assessed using 3-(4,5-dimethyl-2-thiazolyl)-2,5-diphenyl-2-H-tetrazolium-bromide (MTT) assay. Briefly, ARPE-19 cells were washed with 1X PBS and incubated at 37 °C with fresh medium containing 0.5 mg/mL MTT solution (Sigma, M5655). After 4 h, 150 µL DMSO was added to dissolve MTT formazan crystals in each well, and absorbance was measured at 570 nm using a multimode microplate reader (Tecan Infinite 200 PRO M Plex, Morrisville, NC, USA). The viability of ARPE-19 cells was defined as the absorbance of H_2_O_2-_treated cells to the untreated cells. Next, 50–60% confluent cultures of ARPE-19 cells were treated with varying concentrations (25–0.78 µM) of CBD and CBD-EVs for 24 h, and the cell viability was assessed similarly using the MTT assay.

Dichlorofluorescein diacetate (DCFDA) assay was used to assess the intracellular ROS production after 3 h and 6 h of oxidative stress induction with H_2_O_2_ in ARPE-19 cells, which were pre-treated for 24 h with EVs, CBD, or CBD-EVs. Cells not treated with H_2_O_2_ were used as a control. The medium was removed, and cells were washed with 1X PBS. Thereafter, 10 µM DCFDA (Sigma, 21882) reagent was added, and cells were incubated for 30 min at 37 °C in the dark. The cells were washed twice with 1X PBS, and 100 µL of 1X PBS was added to the wells, and images were captured using the fluorescent microscope (Olympus BX51, Center Valley, PA, USA) with a standard FITC filter. Fluorescence intensity was measured and analyzed using ImageJ software (version 1.48, NIH, Bethesda, MD, USA).

### 2.9. Immunofluorescence and Imaging

ARPE-19 cells were seeded at a density of 1.1 × 10^6^ cells/cm^2^ in a 6-well plate. Upon reaching 60–70% confluency, the cells were treated with 300 µM H_2_O_2_ for 24 h. Post 24 h, different formulations of 5 µM CBD, 5 µM CBD-EVs, or EVs were applied to oxidative-stressed ARPE-19 cells for 24 h. Cells not treated with H_2_O_2_ were used as a control. Post 24 h, the cells were fixed with freshly prepared 4% formalin (Sigma, HT501128) for 10 min at 4 °C and further permeabilized with 0.2% Triton X-100 (Sigma, X100) (made in 1X PBS) at room temperature for 20 min. Fixed and permeabilized cells were then blocked with 3% BSA (Sigma, A5611) for 2 h at room temperature, followed by incubation with primary antibody (Supplementary Table S1) overnight at 4 °C. Cells were washed with 1X PBS and stained with fluorescein-conjugated secondary antibodies for 1 h in the dark at room temperature (Supplementary Table S1). Counterstaining with DAPI (Sigma, F6057) for 2 min in the dark at room temperature was applied for nuclear staining. Images were captured using a Zeiss Axio observer Z1 with Axiocam MRm (ZEISS, Thornwood, NY, USA) and analyzed using ImageJ software (version 1.48, NIH, Bethesda, MD, USA).

### 2.10. Western Blotting

Protein lysates of control and different treatment groups of ARPE-19 cells (H_2_O_2_, H_2_O_2_ + 5 µM CBD, H_2_O_2_ + 5 µM CBD-EV) were prepared in radioimmunoprecipitation buffer (RIPA buffer; Sigma Aldrich, R0278) containing 1:100 protease (Sigma Aldrich, P1860) and phosphatase inhibitors (Sigma Aldrich, P0001) [37]. The cell homogenates were centrifuged at 10,000× *g* for 20 min at 4 °C, and the supernatants were collected. The protein content in the sample was estimated using a bicinchoninic acid assay kit (BCA; ThermoFisher Scientific, 23225) as per the manufacturer’s instructions. Briefly, 40 µg of protein samples were loaded and resolved using SDS-PAGE gel electrophoresis and transferred onto the PVDF membrane (Biorad, Hercules, CA, USA;1620177) using the Transblot “Turbo” transfer system (semi-dry transfer unit, BIORAD, Hercules, CA, USA), followed by blocking with 5% BSA solution (made in PBST). After blocking, the PVDF membrane was incubated with primary antibodies diluted in PBST (Supplementary Table S1) at 4 °C overnight. The membranes were then incubated with horseradish peroxidase (HRP)-conjugated secondary antibodies diluted in PBST (Supplementary Table S1) for 2 h at room temperature. The luminescence signal was captured using a ChemiDoc^TM^ XRS^+^ imaging system (BIO-RAD), and the band intensities were quantified using ImageJ software (version 1.48, NIH, Bethesda, MD, USA) [37].

### 2.11. Statistical Analysis

One-way analysis of variation (ANOVA) was performed to compare the significance between groups, followed by post hoc analysis using the recommended Dunnett’s multiple comparison test on the GraphPad Prism software v9.5.0 (GraphPad Software, San Diego, CA, USA). All data were analyzed in consultation with a statistician and were considered statistically significant at *p* < 0.05.

## 3. Results

### 3.1. Cargo Characterization Revealed Distinct DEGs in Late and Early Retinal Organoid EVs

**Figure 2:**
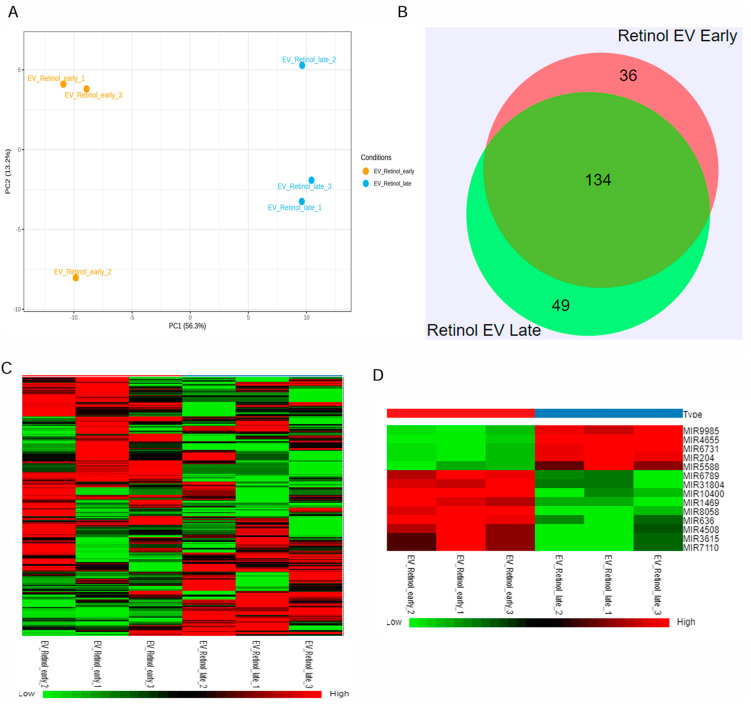
miRNAseq of retinal organoid EVs. (**A**) Principal component analysis (PCA) plots of miRNA-seq showing individual clusters of early and late retinal organoid EVs (n = 3 biological replicates for each). (**B**) Venn diagram showing numbers of miRNAs common between two sample groups and unique for each of the two sample groups, early and late retinal organoid EVs. (**C**) Heatmap showing the log2fold change in DEmiRNAs of all the individual samples with a two-color gradient. Downregulation is indicated in green and upregulation is shown in red. (**D**) The upregulated and downregulated DEmiRNAs for early vs. late retinal organoid EV samples are visually represented as a heat map.

Retinal organoids differentiated from hiPSC via the embryoid body approach were cultured in the 0.1 L PBS-VW bioreactor with 3D-retinal differentiation medium as per the protocol described in our previous studies [34,40]. EVs were isolated from the retinal organoids CCM using a modified differential centrifugation method and PEG precipitation, as reported in our published study [34]. Isolated retinal organoid-EVs expressed tetraspanin markers, including CD63 and CD81, along with Flotillin-2 and Alix [34]. Since small RNA cargo contributes to the therapeutic utility, miRNA-seq was performed in the current study to identify the miRNA cargo in the early and late retinal organoid EVs (Figure 2 and Supplementary Figure S1). The score plot of principal component analysis (PCA) showed a clear separation of three biological replicates from each group of retinal organoid EV samples (Figure 2A). Two different but homogenously tight clusters within each sample group were seen based on the inter- and intra-sample variability for early and late retinal organoid EVs. PCA accounted for 69.5% total variance in the dataset, with 56.3% variance for the first (PC1) and 13.2% variance for the second (PC2) principal components (Figure 2A). While analyzing the miR dataset by Venn diagram, we spotted 219 differentially expressed miRNAs (DEmiRs), of which 134 (~73%) were overlapped between the late and early retinal organoid EVs. Notably, the number of uniquely expressing DEmiRNAs identified in early and late retinal organoid EVs were 36 and 49, respectively (Figure 2B). The top 20 abundant miRNAs identified in the early and late retinal organoid EVs are listed in Table 1. Amongst the top 20 abundant DEmiRNAs, early and late retinal organoid EVs shared 17 common DEmiRNAs, which comprise miR-21, 7, 9, 26a, 92a, 100, 143, 146a, 146b, 148a, 182, and 1246 and let-7f, 7i, 7a, 7g, and 7b. Three distinctly abundant DEmiRNAs contributing to the separation of early retinal organoid EVs were miR-636, 10400, and 8058, while the late retinal organoid EVs expressed miR-5588, 183, and 423 (Table 1). A heat map of DEmiRNAs from two sample groups showed the expression of miRNAs (row clustering) for six individual samples (column clustering) (Figure 2C). When comparing the early to late retinal organoid EV samples, we could distinguish a total of fourteen significantly DEmiRNAs (log2 fold change: Log2FC), encompassing nine upregulated (miR-10400, 636, 8058, 1469, 4508, 6789, 31804, 7110, and 3615) and five downregulated (miR-9985, 4655, 6731, 204, and 5588) genes (Figure 2D and Table 2).

### 3.2. CBD-EVs Structural Properties Supports for Medical Application Use

**Figure 3:**
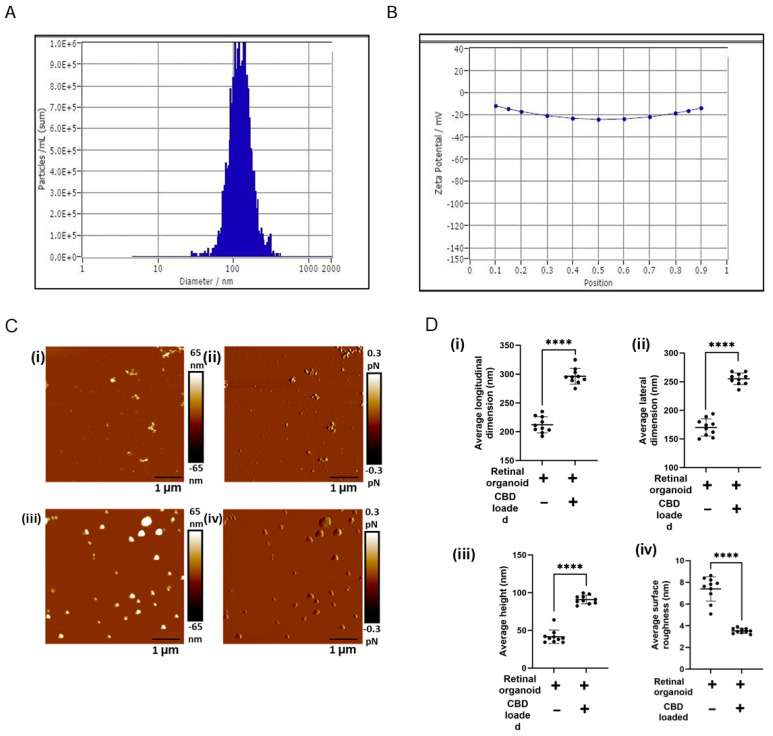
Topography of retinal organoid unloaded EVs and CBD-EVs. (**A**) Representative histogram showing the mean particle size distribution of CBD-EVs by NTA. (**B**) Zeta potential of CBD-EVs. (**C**) Atomic force microscopic images showing the height (i,iii) and peak force error (ii,iv) of non-loaded and CBD-EVs. (**D**) Quantification of unloaded EVs (retinal organoid EVs) and CBD-EVs (CBD-loaded EVs) showing average longitudinal dimension (i), average lateral dimension (ii), average height (iii), and average surface roughness (iv). **** *p* < 0.0001. Scale bar: 1 µm. The color scale represents variations in peak force error and surface texture of particles.

Active internal encapsulation of CBD into EVs via sonication may alter the physical, biophysical, and chemical properties of EVs [30,37]. Hence, we thoroughly characterized the bioengineered EVs (CBD-EVs) according to the minimal information for studies of extracellular vesicles (MISEV) guidelines published by the International Society for Extracellular Vesicles (ISEV) [41]. NTA, a light-scattering method, showed the mean particle size of 120 ± 2.1 nm for EVs encapsulated with 10% CBD (Figure 3A), with an entrapment efficiency of 80 ± 2.42% and a zeta potential of −15.43 ± 0.3 mV (Figure 3B). Our previous in vitro studies showed a sustained release of CBD from exosomes at pH 6.8 and pH 7.4 after 24 h [30]. Since the mechanical properties influence the biological function of EVs, AFM imaging, a qualitative tool, was utilized to investigate the topographical attributes and membrane shape/deformation of retinal organoid EVs with and without CBD encapsulation at a single-particle level. In our preliminary studies (Supplementary Figure S3), AFM was used to assess the changes in the morphology of EVs before and after sonication. From the results obtained, there was no significant change in EV surface morphology after sonication and incubation at 22 °C. A representative AFM image is shown illustrating the height and dimensions (topography) of non-loaded EVs and CBD-EV particles (Figure 3C(i,ii)). The CBD-EV particles appeared larger and brighter than non-loaded EVs, representing them as smaller and darker. The average height of CDB-EVs was measured as 91.26 ± 2.85 nm in comparison to 41.6 ± 8.84 nm in non-loaded EVs, displaying a 2.2-fold elevated height profile in CDB-EVs (Figure 3D(iii)). Additionally, the longitudinal and lateral dimensions of retinal organoid EVs with and without CBD were extracted (Figure 3D(i,ii)). For instance, a significant increase in the longitudinal dimension of CBD-EVs was observed to be 295 ± 13.68 nm, in comparison to non-loaded EVs showing 212 ± 14.07 nm (Figure 3D(i)). Conversely, the lateral dimension of CBD-EVs was also higher at 254.44 ± 10.4 nm, whereas the non-loaded EVs were observed as 170.2 ± 15.09 nm (Figure 3D(ii)). Next, a qualitative AFM image showing the peak force error was visualized on a separate channel alongside the topography image to obtain insight into variations in the feedback signal loop between the tip-sample interactions and to measure the particle’s surface features (Figure 3C(iii,iv)). A particle’s surface variation, such as smoothness or roughness, is also a measure of topographical features. As seen in Figure 3D(iv), surface roughness is significantly decreased to 3.56 ± 0.2 nm in CBD-EVs, making the overall topography smoother compared to the rough non-loaded EVs with 7.39 ± 1.12 nm. These morphological attributes confirmed the therapeutic use of mediating intercellular communicating characteristics for CBD-EVs.

### 3.3. CBD-EVs Activate AMPK Signaling and Mitigate Apoptosis in Mitochondria-Induced Oxidative ARPE-19 Cells

**Figure 4:**
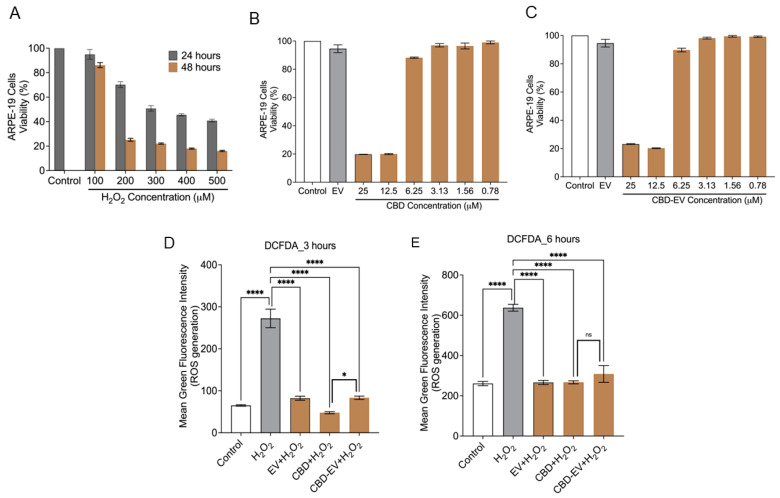
Cytotoxicity and ROS assay in ARPE-19 cells. (**A**) ARPE-19 cell viability measured using the MTT assay after treating with H_2_O_2_ (100, 200, 300, 400, 500 µM) for 24 and 48 h. ARPE-19 cells not exposed to H_2_O_2_ are shown as a control. (**B**,**C**) Cell viability assessment using the MTT assay for ARPE-19 cells treated with different concentrations (25, 12.5, 6.25, 3.12, 1.56, 0.78 µM) of either CBD (**B**) or CBD-EVs (**C**) for 24 h. ARPE-19 cells not treated with H_2_O_2_ are shown as a control. ARPE-19 cells treated with EVs but not H_2_O_2_ are also shown as a control. (**D**,**E**) Measurement of ROS using DCFDA staining in 24 h pretreated live ARPE-19 cells with various antioxidant formulations (EV, CBD, and CBD-EV) and further treated with H_2_O_2_ for oxidative damage at 3 h (**D**) and 6 h (**E**). Bar graphs represent the ROS generation measured in relative mean green fluorescence intensity. Statistical one-way analysis, Dunnett test with 95% confidence level. N = 3 independent replicates. All the data are represented as ± SED, **** *p* < 0.0001, * *p* < 0.05 compared to H_2_O_2_. ns = non-significant; DCFDA = dichlorofluorescin diacetate; MTT = 3-(4,5-dimethylthiazol-2-yl)-2,5-diphenyltetrazolium bromide assay; ROS = reactive oxygen species.

Cell death due to oxidative stress in the RPE and retina (photoreceptors) is hypothesized to play an essential role in the pathogenesis of AMD. Therefore, the apoptotic effect of oxidative stress was examined in ARPE-19 cells treated with different concentrations of H_2_O_2_ (100–500 µM) for 24 and 48 h. MTT assay results showed that the cell viability was reduced to ~50% at 24 h treatment when exposed to 300 µM of H_2_O_2_ in comparison to the control (untreated H_2_O_2_) (Figure 4A). H_2_O_2_-induced ARPE-19 cells were seen to be both dose- and time-dependent. Based on this result, ARPE-19 cells treated with 300 µM H_2_O_2_ for 24 h were chosen to induce apoptosis and ROS production for further experiments. Next, we sought to evaluate the toxicity of CBD and CBD-EVs on ARPE-19 cells. Upon 50–60% confluency, ARPE-19 cells treated with CBD or CBD-EVs for 24 h showed >80% viability when 6.25–0.78 µM concentration was applied to the cells, signifying no signs of apoptosis in ARPE-19 cells (Figure 4B,C). ARPE-19 cells that were not treated with H_2_O_2,_ CBD, or CBD-EVs were used as a control. The viability of ARPE-19 cells remained unchanged when treated with 1 mg/mL of unloaded EVs. Based on our data, we chose to use 5 µM CBD or CBD-EVs for assessing the ROS production by DCFDA cell-based assay. To investigate the antioxidant potential, we pretreated 70% confluent cultures of ARPE-19 cells with 1 mg/mL EVs, 5 µM CBD, and 5 µM CBD-EVs for 24 h and further exposed the cells to H_2_O_2_ (3 h and 6 h) to assess for the intracellular ROS production. All three formulations significantly prevented ROS production when induced with H_2_O_2_ stress at both 3 h and 6 h, confirming their antioxidant characteristics (Figure 4D,E). There were no significant differences within the EV, CBD, and CBD-EV formulations. Cultured ARPE-19 cells and H_2_O_2_-induced ARPE-19 cells were used as negative and positive controls, respectively.

**Figure 5:**
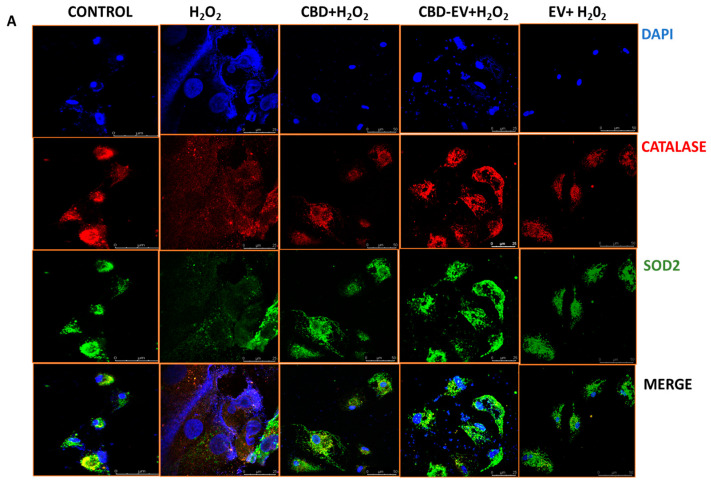

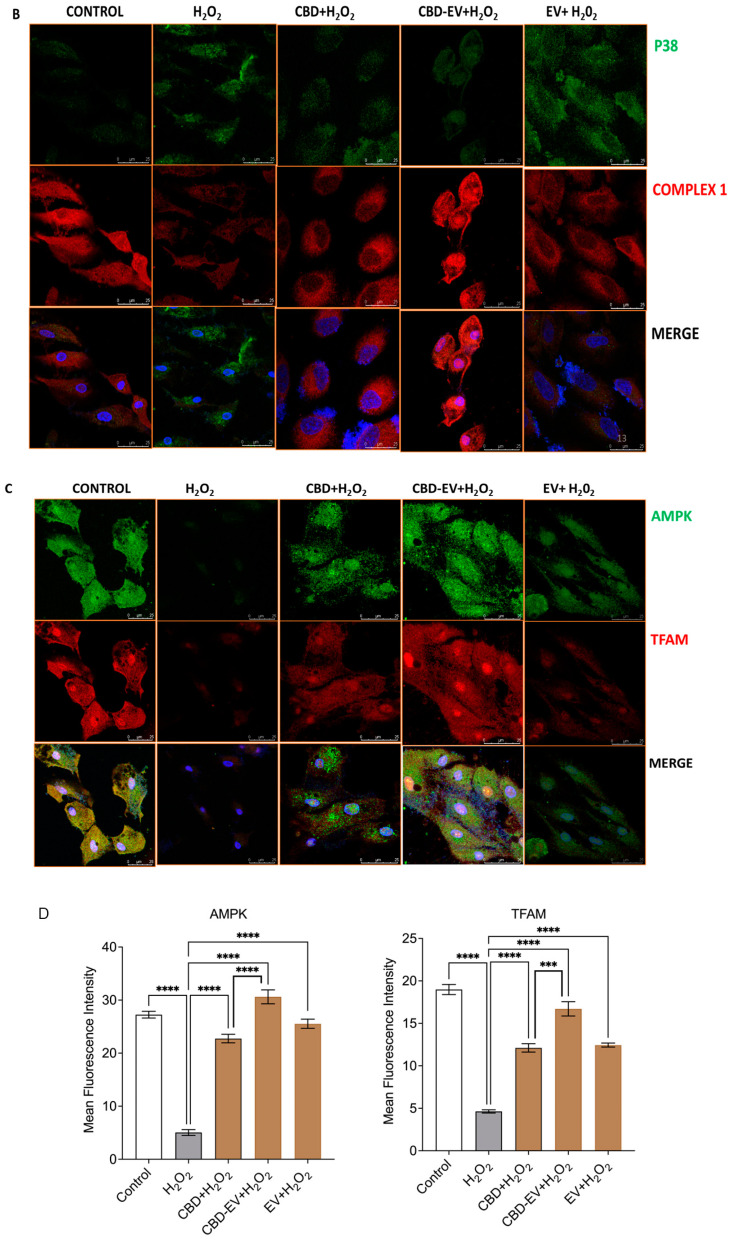

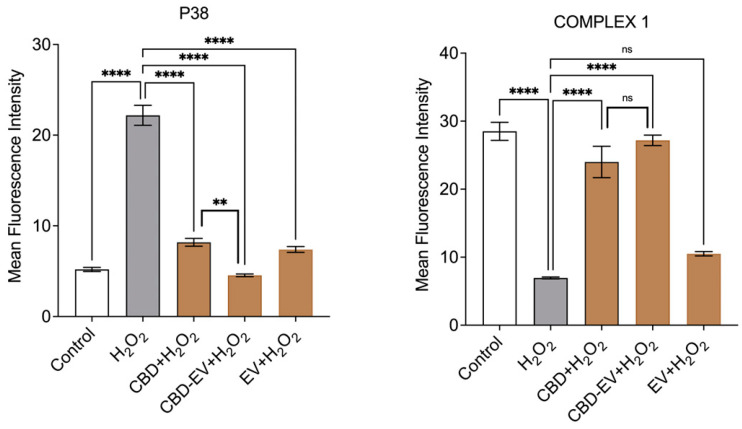
Immunostaining of oxidatively damaged ARPE-19 cells. Representative confocal microscope images immunoreactive for (**A**) antioxidant proteins, CATALASE (red) and SOD2 (green); (**B**) apoptosis protein marker, P38 (green), and mitochondrial respiratory COMPLEX 1 (red); (**C**) anti-oxidative mitochondrial-stress tolerance markers, AMPK (green) and TFAM (red). DAPI counterstain for all nuclei (blue). Scale bar = 25 µm. (**D**) Mean fluorescent intensity of the immunofluorescent images stained for AMPK, TFAM, P38, and COMPLEX1. Statistical one-way analysis, Dunnett test with 95% confidence level. N = 3 independent replicates. All the data are represented as ± SED, **** *p* < 0.0001; *** *p* < 0.001; ** *p* < 0.01, ns = non-significant, compared to H_2_O_2_. CBD + H_2_O_2_ were also compared with CBD-EVs + H_2_O_2_.

Furthermore, immunofluorescence staining with a few known classical protein markers for AMD, such as antioxidant markers (catalase and SOD2), mitochondrial function-related proteins (complex 1, TFAM, and AMPK), and a stress-signaling apoptosis protein (p38), was performed to validate the oxidative stress mechanism (Figure 5). Immunoreactivity to SOD2 and catalase was strongly evident in control and H_2_O_2_-induced ARPE-19 cells treated with CBD, CBD-EVs, and EVs, demonstrating their protective antioxidant effect against mitochondrial damage and apoptosis. In contrast, H_2_O_2_-induced ARPE-19 cells clearly showed weaker expression of SOD2 and catalase, displaying an altered mitochondrial transmembrane potential (MTP) with a lack of the cells’ ability to detoxify oxidative stress. Moreover, the swelling of the cell nuclei shown in blue by DAPI staining confirmed the loss of cell viability in H_2_O_2_-induced ARPE-19 cells (Figure 5A). To further clarify the MTP, we assessed the functioning of mitochondrial respiration by staining with complex 1 and found weak expression in H_2_O_2_-induced ARPE-19 cells. This deficiency was reversed with a dramatic increased expression of complex 1 in the groups of ARPE-19 cells treated with CBD, CBD-EVs, and EVs (Figure 5B,D). Complex 1-reactive ARPE-19 cells were detected at low levels in the EVs group, albeit not statistically significant concerning the control, and clearly implicating the advantages of CBD encapsulation into the EVs (Figure 5D; *p* < 0.0001). Notably, intense immunoreactivity of AMPK- and TFAM-positive ARPE-19 cells was also apparent in the control, CBD-, CBD-EV-, and EV-treated groups (Figure 5C). A significantly elevated expression of TFAM, a mitochondrial DNA transcription factor A, suggested the reduction in mitochondrial-induced oxidative cell death (Figure 5D; *p* < 0.001). These data supported the notion that dynamic activation of AMPK signaling is imperative to maintain homeostasis and in alleviating the metabolic stress of high-energy-consuming cells.

Finally, a class of mitogen-activated protein kinase (MAPK), p38, is known to be responsive to stress stimuli and is an established marker of cell apoptosis [42].Therefore, fluorescent staining of H_2_O_2_-induced ARPE-19 cells exposed to various antioxidant formulations (CBD, CBD-EV, EV) with p38 showed no or significantly reduced expression, almost to the levels of the control when compared to ARPE-19 cells treated with H_2_O_2_ (Figure 5B,D; *p* < 0.001).

### 3.4. CBD-EVs Provide AMPK-Dependent Protective Effects in H_2_O_2_-Treated ARPE Cells

**Figure 6:**
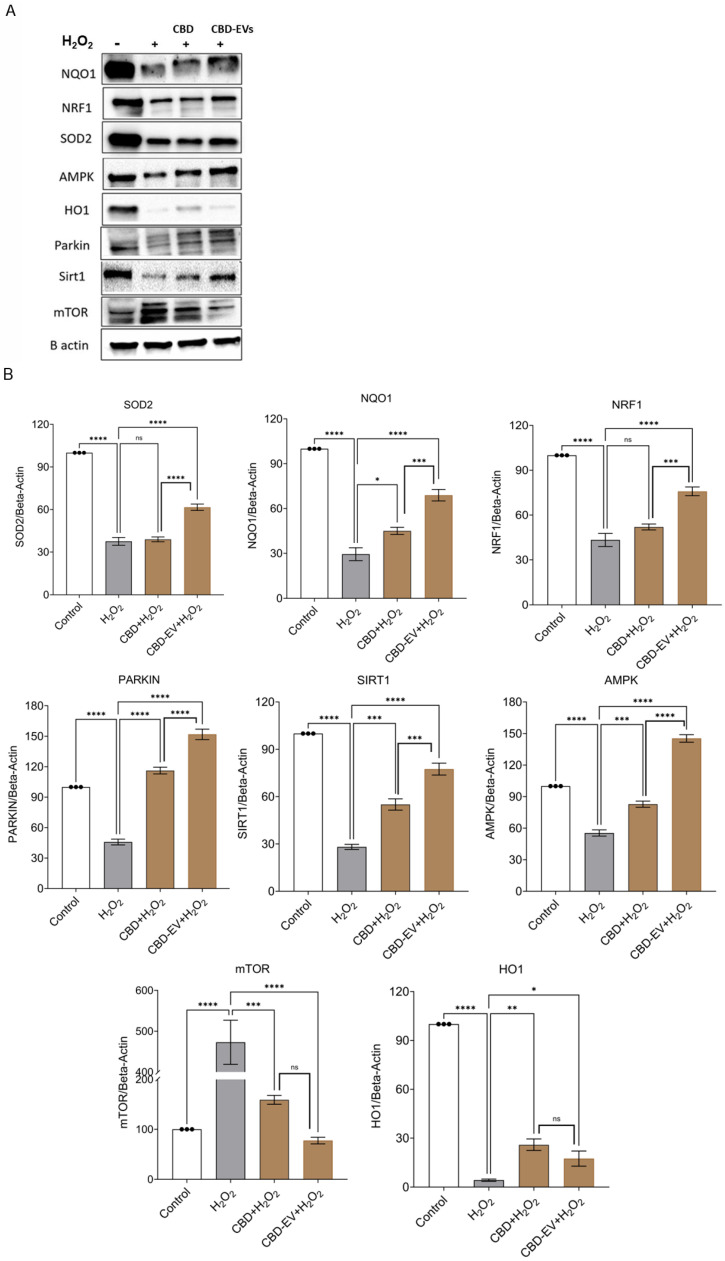
Western blotting analysis of CBD and CBD-EV regulating proteins. ARPE-19 cell lysates stressed with H_2_O_2_ (300 µM) and further treated with CBD (5 µM) and CBD-EVs (5 µM). (**A**) Representative immunoblots of total protein levels probed against ß-actin (internal control), antioxidant, and AMPK-SIRT1-NRF1/2 signaling proteins (NQO1, SOD2, HO1, PARKIN, mTOR, NRF1, AMPK, SIRT1). (**B**) Quantitative analysis of targeted proteins normalized to ß-actin and relative to control. Statistical one-way analysis, Dunnett test with 95% confidence level. N = 3 independent replicates. All the data are represented as ± SED, * *p* < 0.05, ** *p* < 0.01, *** *p* < 0.001, **** *p* < 0.0001; ns = non-significant, compared to H_2_O_2_. CBD + H_2_O_2_ were also compared with CBD-EVs + H_2_O_2_. HO1 = heme Oxygenase-1; mTOR = mechanistic target of rapamycin; NQO1 = NAD(P)H:quinone oxidoreductase 1; NRF1 = NF-E2-related factor 1; SOD2 = superoxide dismutase.

To corroborate the immunohistochemistry results, a Western blot assay was performed to determine the protective effects of CBD and CDB-EV in oxidative-stressed ARPE-19 cells. The findings show a significant downregulation of mTOR (*p* < 0.0001) and significant upregulation of SOD2 (*p* < 0.0001), PARKIN (*p* < 0.0001), SIRT1 (*p* < 0.0001), NQO1 (*p* < 0.0001), NRF1 (*p* < 0.001), HO-1 (*p* < 0.05), and AMPK (*p* < 0.0001) in the lysates of ARPE-19 cells treated with CBD-EVs in comparison to H_2_O_2_ groups (Figure 6A,B). While CBD also showed similarly significant results when compared to CBD-EVs, with the exception of mTOR and HO-1 being non-significant. All these targets have been proposed as a plausible underlying mechanism of CBD-mediated neuroprotection, as shown in Supplementary Figure S2.

## 4. Discussion

CBD has been shown to play an essential role in mitigating oxidative stress as a direct antioxidant (Supplementary Figure S2) [43,44]. However, the stability of CBD has impeded their use as a therapeutic agent due to their oxidative properties. Previous studies from our lab had developed CBD-EV formulations using mesenchymal stem cell-derived EVs to overcome this limitation, and their application has been reported to be effective in treating athymic nude mice with triple-negative breast cancer and paclitaxel-induced neuropathic pain (PIPN) [30,37]. Characterization of EVs is essential for understanding the mechanistic role in tissue homeostasis and disease pathophysiology, as well as for diagnostic and therapeutic applications. AFM is a label-free surface characterization technique capable of providing both qualitative and quantitative morphological attributes of numerous biological samples, including EVs [45]. This technique preserves the structural integrity and has proven to be advantageous over other traditionally used techniques such as transmission electron microscopy (TEM) and scanning electron microscopy [46]. Various studies have demonstrated the therapeutic utility of exosomes as nanocarriers for drug delivery systems; henceforth, a comprehensive structural, molecular, and biomechanical characterization is pivotal [47]. Prior studies on malignant melanoma (MM) had used AFM to measure the size of exosomes derived from the primary cancer stem cell lines of MM patients and compared them with the serum-derived exosomes of healthy patients. Two-dimensional images of AFM showed differences such as heterogeneous organization of exosome size and shape regardless of their origin [48]. One of the studies on the breast cancer paradigm has demonstrated AFM’s ability to detect tissue factor (TF)-positive exosomes in patients [49]. Authors showed that the size of the TF-exosomes released by MDA-MB-231 cells varied between 20–60 nm and 110–652 nm when imaged in air and liquid mode, respectively. The characterization of vesicle membrane expression with antibodies specific to TF in the liquid mode of AFM precisely resembles the physiological conditions in the cancer paradigm. Such applications have not been explored in the AMD paradigm yet.

Furthermore, determining the surface roughness characteristics suggests the topographical variations occurring due to the encapsulation/loading of drug on the EVs. A research study explored the benefits of using AFM tools to screen the EVs derived from the human neural stem cell line, CTX0E03, ascertaining biochemical changes. In this study, the authors adopted different techniques, such as ultracentrifugation, sonication, and AFM, to identify the subtle differences in the structural and mechanical properties of EVs. EVs prepared using the ultracentrifugation technique had slightly increased physical dimensions, decreased adhesion, and lower CD63 expression levels. Conversely, the sonicated EVs showed a reduction in both the size and adhesive force with reduced CD81 expression [50]. Nonetheless, the effect of these phenotypic characterizations of EVs in therapeutic applications was not demonstrated. By combining the NTA and AFM data, we noticed that the CBD-encapsulated retinal organoid EVs had a significantly increased height profile. The biomechanical properties of EVs are not easily discernible using TEM or any other previously mentioned techniques. The data obtained through the AFM technique distinctly mimics the biological fluid environment. Unlike studies performed in an air medium using TEM, where EVs are dried and lose their biomechanical properties, AFM preserves the actual shape and biomechanical properties by capturing the phenotype in the liquid mode [49,51]. As reported in our earlier studies, EVs express definite endosomal pathway markers, including tetraspanin (CD63 and CD81), heat shock proteins (HSP70), and Rab family proteins such as TSG101 and Alix [34]. Calnexin was not expressed by EVs, and our previous study had clearly established the expression of all the definite markers in both the early and late retinal organoid EVs [34].

In our earlier research, we reported the successful isolation of EVs by differentiating 3D retinal organoids from human iPSCs via the embryoid-body approach and by the long-term maintenance of the organoids in a PBS-Vertical Wheel (PBS-VW) bioreactor [34]. We profiled and compared the EVs of hiPSC-differentiated 3D retinal organoids with human umbilical cord mesenchymal stem cell (hUCMSC)-derived EVs at the biophysical, nanostructural, nanomechanical, molecular, and proteomic levels. The proteome profile of EVs secreted by retinal organoids was highly indicative of the proteins involved with retinal function as well as EV biogenesis. The presence of retinal marker proteins in retinal organoid EVs will potentially provide a clinically robust therapeutic effect on use in retinal diseases more likely than hUCMSC-EVs. These EVs are continuously released during the retinal organoids’ development, with an increasing expression of EV marker proteins including HRS, Alix, Caveolin-1, HSP70, Flotillin-2, and CD63 from older retinal organoids (>90 days) than in younger retinal organoids (<90 days) [34]. Moreover, our study also assessed the small RNA profile in the early and late retinal organoid EVs.

Micro RNAs (miRs) are an appropriate target for developing therapeutics and biomarkers since they are crucial in regulating RPE physiology and disease [52]. The RPE’s growth and function are controlled and influenced by miRs. Numerous tissues and organs have shown age-related alterations in the miRNA profiles [53,54,55], significantly impacting the critical components of aging-related signaling pathways [56]. Exosome miRNAs are determined to orchestrate the molecular signaling pathways via intra- and inter-cell-to-cell communications [57,58]. MicroRNAs that were commonly expressed in both the early and late retinal organoid groups of EVs were miR-21, let-7f, 7i, 7a, 7g, 7b, miR-7, 9, 26a, 92a, 100, 143, 146a, 146b, 148a, 182, and 1246. Three unique and distinct microRNAs identified in the late retinal organoid group included miR-183, 423, and 5588. Interestingly, in our study, the heatmap analysis revealed the distinction of significantly upregulated miRNAs in the late retinal organoid group, which included miR-4655, 6731, 204, 9985, and 5588. Due to the enriched miRNA cargo in the late retinal organoid-EVs, these were utilized for CBD encapsulation and validated in our current in vitro model of oxidative-stressed ARPE-19.

Studies have shown that miR-21 can modify the function of retinal endothelial cells by regulating specific signaling proteins, such as tissue inhibitors of matrix metalloproteinases-3 [59] or peroxisome proliferator-activated receptor [60]. The retina’s inner nuclear layer (INL) and the mature photoreceptors highly express the miRNA cluster, miR-182/96/183 [61]. According to a study by Busskamp et al. (2014), miR-182 and miR-183, as well as miR-96, are particularly crucial for the sustenance and functionality of cone outer segments [62]. The cluster is generally responsible for the global regulation of numerous downstream genes involved with synaptogenesis, synaptic transmission, and photoreceptor functions [62]. Therefore, miR-183 was identified to be expressed in only late-matured retinal organoid EVs and not in early retinal organoid EVs. Studies have shown that some miRNAs, such as let-7, are involved in the development of specific cell types and the evolution of retinal progenitor cells (RPCs) [63,64]. According to a study by Xia et al. (2016), let-7 likely promotes the differentiation of Müller glia and neuronal cells by suppressing the expression of Hmga2, a DNA architecture protein involved in the self-renewal of neural progenitors [65].

miR-204 is highly expressed in RPE cells and to some extent in the neural retina, including the ciliary body, epithelial cells of the lens, ganglion cell layer, and the trabecular meshwork [66]. miR-204 plays a vital role in the maintenance and functionality of photoreceptors in the retina [67,68]. It is worth noting that our miRNA sequencing results identified miR-204 in both early and late retinal organoid EVs, although at significantly higher levels in the late retinal organoid EV group. Indeed, our current study data correlates with a study by Zhou et al. (2021), where they had characterized the different developmental stages of retinal organoid-derived EVs using miRNA transcriptome approaches [69]. The miRNA cargo identified in the different developmental time points of retinal organoid EVs was allied to the in vivo retinogenesis event for the targeted genes [69].

To the best of our knowledge, this is the first study to provide a proof of concept by demonstrating the therapeutic effect of CBD-loaded retinal organoid EVs in vitro. Extensive research has been conducted on AMD mechanisms, including increased oxidative stress, altered mitochondrial bioenergetics, dysregulated RPE metabolic metabolism, and increased inflammation [6,70]. These findings suggest that regulating metabolic activity may improve rod, cone, and RPE survival and function in patients with retinal degenerations. AMPK is a cellular energy sensor activated upon nutrient deprivation, low energy states, fasting, or hypoxia [71,72]. A study by Xu et al. (2018) demonstrated that AMPK activators can provide broad-spectrum protection in the retina, preventing vision loss from an acute injury in inherited retinal degeneration, including age-related macular degeneration [73]. The results from Xu et al.’s (2018) study show that metformin, an AMPK activator, protected photoreceptors from acute light damage, delayed retinal degeneration, and protected the RPE from oxidative stress-induced injury [73].

The use of hUCMSC-EVs could alleviate myocarditis in the in vitro- and in vivo-induced models of myocarditis and improve cardiac function by increasing AMPK activity, promote the degradation of autophagy flux proteins, and downregulate apoptosis proteins [74]. Our previous reports on PIPN show that the administration of CBD-EVs formulation increased p-AMPK (Thr 172) expression in PTX-treated mice dorsal root ganglion (DRG) homogenates and DRG neuronal cells [37]. In this study, H_2_O_2_-treated ARPE-19 cells also showed lower SIRT1 expression compared to the normal control group. When AMPK is activated, it increases intracellular NAD^+^ and activates SIRT1, deacetylating and translating NRF1 and NRF2 into the nucleus. We observed the similar pattern of increased SIRT1 protein expression in our study when oxidatively stressed ARPE-19 cells were treated with CBD and CBD-EVs. In human neuroblastoma cells (in vitro Parkinson model), CBD activated the SIRT1 pathway, increasing the autophagy and mitochondrial activity [37]. Supplementary Figure S2 proposes a plausible underlying mechanism of cannabinoid-mediated neuroprotection. Amongst these potential targets, SIRT1 plays a crucial role in protecting mitochondrial dysfunction in neuronal cells via the cannabidiol-TRPV1 pathway. Upregulation of SIRT1 also inhibits NF-κB and NOTCH pathways, thereby inducing autophagy to protect against mitochondrial dysfunction in neuronal and SH-SY5Y cells [75]. Our previous studies have shown that CBD and CBD encapsulated in MSC-derived EVs mitigate the PIPN by modulating the AMPK and mitochondrial function in C57BL/6J mice [37]. Protein expression of HO-1 (*p* < 0.05) and mTOR (*p* < 0.001) was significantly lower in the CBD-EVs group in comparison to the H_2_O_2_ group (Figure 6A,B). Autophagy is known to be induced under stress by mTOR inhibition [76]. Although AMPK and mTOR tend to regulate distinct pathways, there are important crosstalk hubs where both converge to control homeostatic functions like cell metabolism and autophagy. Different targets are employed by AMPK to efficiently decrease mTOR Complex 1 (mTORC1). AMPK phosphorylates Raptor, which in turn inhibits mTORC1 directly [77]. This explains the correlation between the upregulation of AMPK expression and the downregulation of mTOR in the CBD-EVs group.

Golestaneh et al. recently developed an in vitro disease model of AMD by reprogramming RPE to iPSCs from patients with AMD and differentiating these iPSCs back to RPE (AMD: RPE-iPSC-RPE). SIRT1 and PGC-1 were downregulated in AMD: RPE-iPSC-RPE compared to normal RPE-iPSC-RPE [78]. According to the findings, dysfunctional SIRT1/PGC-1 may reduce mitochondrial activity and increase the reactive oxygen species (ROS) production in AMD: RPE-iPSC-RPE, contributing to AMD pathophysiology [78]. Furthermore, AMPK phosphorylation activates a cascade of downstream proteins, including NRF1/2, for regulating mitochondrial biogenesis and function. Additionally, the transcriptional activity of NRF1/2 triggers the TFAM in neuronal cells for the maintenance and transcription of mitochondria [44]. In the current study, we found CBD and CBD-EV formulations increased the expression of SOD2, NQO1, and HO1 in the oxidative stress model of ARP-19 cells. In addition to regulating mitochondrial bioenergetics, AMPK activates NRF2 and NF-κB signaling pathways, mainly to regulate oxidative stress and inflammation [79]. To validate the activation of the AMPK pathway driven by CBD and CBD-EVs, future studies employing the use of a specific AMPK inhibitor can confirm the mechanism and lay down the foundation for potential clinical use.

## 5. Conclusions

In summary, the primary finding of this study establishes the proof-of-principle for CBD-encapsulated retinal organoid EVs to have a superior antioxidant effect in an oxidative stress model of ARPE-19 cells. Retinal organoid EVs serve as a drug delivery carrier, and CBD/CBD-EVs have protective therapeutic effects on ARPE-19 cells by targeting precise proteins linked to the activation of the AMPK pathway. Retinal organoids release EVs throughout the development, and these EVs express miRNAs that are crucial for retinal function, development, and maintenance. Administering encapsulated CBD to retinal organoid EVs can be a potential therapeutic strategy to prevent the progression and emergence of AMD. Further in vivo preclinical studies are needed to establish the usefulness of retinal organoid EVs and CBD-EVs.

## Figures and Tables

**Table 1:** List of top 20 abundant miRNAs expressed in the early and late retinal organoid EV groups.

EV_Retinal_Early	EV_Retinal_Late
has_miR-21	has_miR-21
has_miR-146b	has_let-7f
has_let-7f	has_let-7i
has_let-7a	has_miR-9
has_let-7i	has_let-7a
has_let-7g	has_miR-1246
has_miR-100	has_miR-146b
has_miR-636	has_miR-100
has_miR-143	has_let-7g
has_miR-1246	has_miR-7
has_miR-7	has_miR-26a
has_miR-10400	has_miR-143
has_miR-9	has_miR-92a
has_miR-92a	has_miR-148a
has_miR-26a	has_miR-182
has_let-7b	has_miR-5588
has_miR-146a	has_let-7b
has_miR-148a	has_miR-183
has_miR-182	has_miR-146a
has_miR-8058	has_miR-423

**Table 2:** The upregulated and downregulated DEmiRNAs for early vs. late retinal organoid EV samples as shown in Figure 2D. Downregulation is indicated in green and upregulation is shown in red.

Name	Log_2_FC	Adj.P. Val
miR-4655	5.26	3.8 × 10^−4^
miR-6731	4.36	3.8 × 10^−4^
miR-204	4.26	3.8 × 10^−4^
miR-9985	3.59	8.9 × 10^−4^
miR-5588	1.94	3.2 × 10^−4^
miR-3615	−2.09	4.7 × 10^−2^
miR-7110	−2.09	4.7 × 10^−2^
miR-31804	−2.21	1.8 × 10^−2^
miR-6789	−2.46	1.1 × 10^−2^
miR-4508	−3.89	1.1 × 10^−2^
miR-1469	−4.45	8.9 × 10^−4^
miR-8058	−5.74	3.8 × 10^−4^
miR-636	−5.98	8.9 × 10^−4^
miR-10400	−6.01	3.8 × 10^−4^

## Data Availability

The original contributions presented in this study are included in the article/Supplementary Materials. Further inquiries can be directed to the corresponding author.

## Supplementary Materials

The following supporting information can be downloaded at: https://www.mdpi.com/article/10.3390/biomedicines13051167/s1, Figure S1: The number of the identified miRNAs by the miRNA-sequencing; Table S1: Primary and secondary antibodies; Figure S2: Plausible mechanism of action of cannabinoids to protect peripheral neurons; Figure S3: Topographical measurements by atomic force microscopy shows EVs remain unchanged after sonication. Reference [43] are cited in the supplementary materials and Figure S2 was reproduced from Arthur et al., (2024) [43] under a Creative Commons License 1605589-1.

## Abbreviations

The following abbreviations are used in this manuscript:

AMD	Age-related macular degeneration
EV	Extracellular vesicle
miRNAs	Micro RNAs
RPE	Retinal pigment epithelium
SASP	Senescence-associated secretory phenotype
CLX	CBD-loaded-exosome delivery platform
PTX	Paclitaxel
CCM	Culture-conditioned medium
PBS	Phosphate-buffered saline
PDA	Photodiode array
CBD	Cannabidiol
hiPSC	Human induced pluripotent stem cell
AMPK	Adenosine monophosphate kinase pathway
ROS	Reactive oxygen species
NMDA	N-methyl-D-aspartate
hUCMSC	Human umbilical cord mesenchymal stem cell
DRG	Dorsal root ganglion
NTA	Nanoparticle tracking analysis
AFM	Atomic force microscope
MTT	3-(4,5-dimethyl-2-thiazolyl)-2,5-diphenyl-2-H-tetrazolium-bromide
DCFDA	Dichlorofluorescein diacetate
MISEV	Minimal information for studies of extracellular vesicles
ISEV	International society for extracellular vesicles
MAPK	Mitogen-activated protein kinase
PIPN	Paclitaxel-induced neuropathic pain
DRG	Dorsal root ganglion
