# Supplementary material for: Cannabidiol-Loaded Retinal Organoid-Derived Extracellular Vesicles Protect Oxidatively Stressed ARPE-19 Cells
Source: Biomedicines. 2025 May 10;13(5):1167. doi: 10.3390/biomedicines13051167 (PMC12108686; doi:10.3390/biomedicines13051167)
Supplement: Supplementary file 1 [file biomedicines-13-01167-s001.zip › biomedicines-3590957-supplementary.pdf]

Cannabidiol-Loaded Retinal Organoid-Derived Extracellular Vesicles Protect Oxidatively Stressed ARPE-19 Cells

Supplementary Materials

Peggy Arthur [1], Sangeetha Kandoi [2,3,4], Anil Kalvala [1], Breana Boirie [1], Aakash Nathani [1], Mounika Aare [1], Santanu Bhattacharya [5,6], Tanmay Kulkarni [5], Li Sun [7,8], Deepak A. Lamba [2,9], Yan Li [7,*] and Mandip Singh [1,*]

Supplementary Figure S1. The number of miRNAs identified using miRNA sequencing.

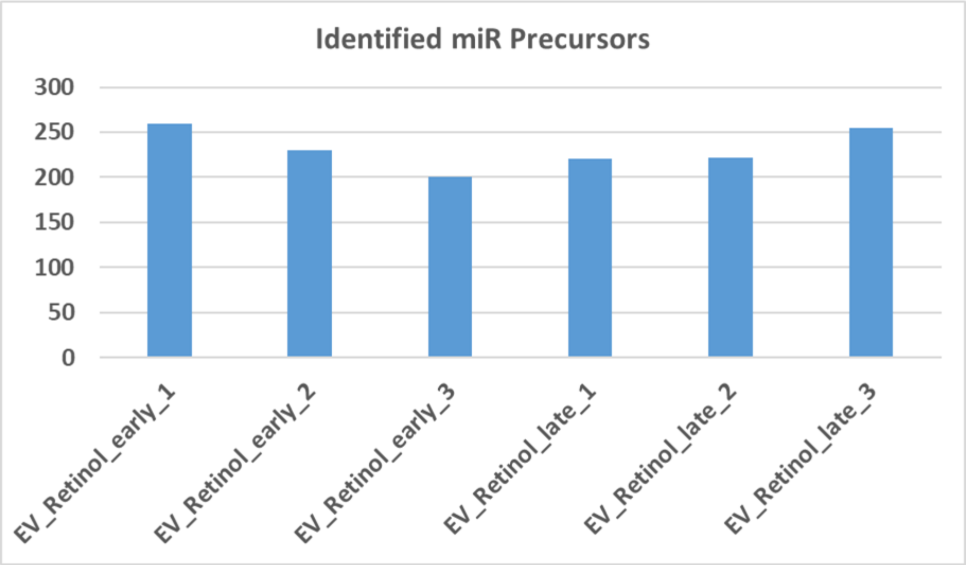

Supplementary Table S1. Primary and secondary antibodies.

**Protein Name**	**Host**	**Source**	**Catalog number**	**Dilution**
AMPK	Mouse	Santa Cruz Biotechnology	SC-398861	1:1000 (Western blotting)<br>1:200 (Immunofluorescence)
Beta-actin	Rabbit	Cell Signaling Technology	4970S	1:1000
Catalase	Mouse	Santa Cruz Biotechnology	271803	1:200
Complex 1	Mouse	Invitrogen	43-8800	1:200
HO1	Rabbit	Cell Signaling Technology	43966S	1:1000
mTOR	Rabbit	Cell Signaling Technology	2972	1:1000
NRF1	Rabbit	Cell Signaling Technology	46743S	1:1000
NQO1	Mouse	Santa Cruz Biotechnology	SC-32793	1:1000
Parkin	Mouse	Santa Cruz Biotechnology	SC-32282	1:1000
P38	Rabbit	Cell Signaling Technology	9212	1:200
Sirt1	Rabbit	Cell Signaling Technology	9475S	1:1000
SOD2	Rabbit	Cell Signaling Technology	13141S	1:1000 (Western blotting)<br>1:200 (Immunofluorescence)
TFAM	Mouse	Santa Cruz Biotechnology	166965	1: 200

Mouse anti-rabbit IgG-CFL 488	Mouse	Santa Cruz Biotechnology	516248	1:200 (Immunofluorescence)
Rabbit anti-mouse IgG-PE	Rabbit	Santa Cruz Biotechnology	358926	1:200 (Immunofluorescence)
Rabbit anti-mouse IgG-HRP	Rabbit	Santa Cruz Biotechnology	358914	1:5000 (Western blotting)
Mouse anti-rabbit IgG-HRP	Mouse	Santa Cruz Biotechnology	2357	1:5000 (Western blotting)

Supplementary Figure S2. Plausible mechanism of action of cannabinoids to protect peripheral neurons. Cannabinoids have been found to activate TRPV1 ion channels, GPR55, and 5HT1A receptors, including CB1 and CB2 receptors. Upon activation, these receptors regulate the downstream AMPK, Nrf2, NF-κB, and BDNF cell signaling pathways. All these orchestrated events collectively improve the neurogenesis, increase the autophagosome formation, augment mitochondrial biogenesis and function, and enhance the antioxidant response for decreasing the inflammation in different conditions of neuropathy[1]. AMPK: adenosine monophosphate-activated protein kinase; Atg: anti-thymocyte globulin; BDNF: brain-derived neurotrophic factor; FIP200: FAK family kinase-interacting protein of 200 kDa; GSH: glutathione; HO1: heme oxygenase 1; mTOR: mechanistic target of rapamycin; SIRT1: silent mating type information regulation 2 homolog 1; NRF: nuclear respiratory factor; Nrf2: nuclear factor erythroid 2 (NFE2)-related factor 2; NF-κB: nuclear factor kappa-light-chain-enhancer of activated B cells; PGC-1α: peroxisome proliferator-activated receptor gamma coactivator 1-alpha; TFAM: mitochondrial transcription factor; Ulk1: unc-51 like autophagy activating kinase; VPS34: vacuolar protein sorting.

Figure reproduced from Arthur et al. **(2024) under a Creative Commons License**

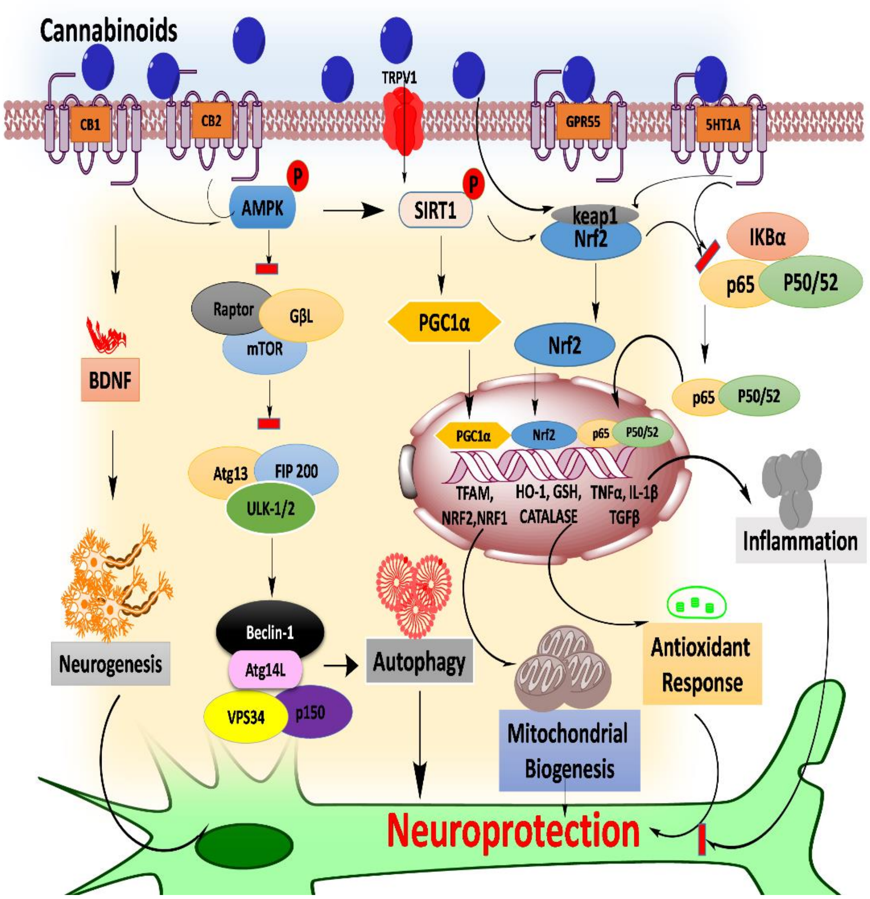
Cannabinoids
CB1
CB2
TRPV1
GPR55
5HT1A
AMPK
P
SIRT1
P
keap1
Nrf2
IKBα
p65
P50/52
Raptor
GβL
mTOR
PGC1α
Nrf2
BDNF
p65
P50/52
PGC1α
Nrf2
p65
P50/52
Atg13
FIP 200
ULK-1/2
TFAM,
NRF2,NRF1
HO-1, GSH,
CATALASE
TNFα, IL-1β
TGFβ
Inflammation
Neurogenesis
Beclin-1
Atg14L
VPS34
p150
Autophagy
Antioxidant
Response
Mitochondrial
Biogenesis
Neuroprotection

Supplementary Figure S3. Topographical measurements by atomic force microscopy show EVs remain unchanged after sonication. Quantification of retinal organoid EVs and sonicated EVs showing average longitudinal dimension (i), average lateral dimension (ii), average height (iii), and average surface roughness (iv).

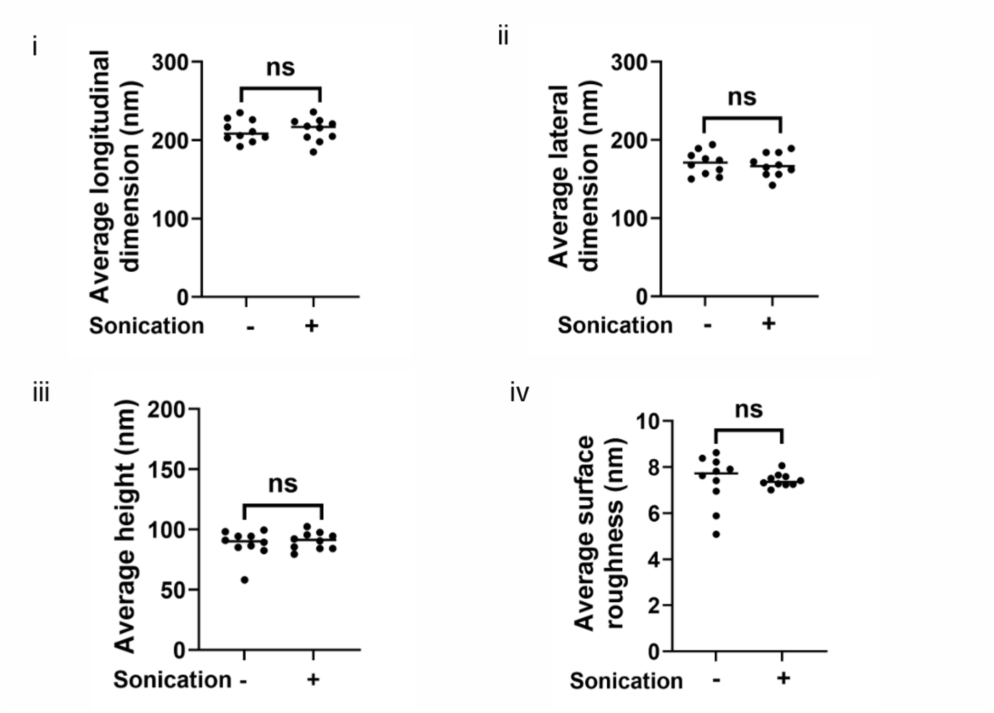

Reference

[1] P. Arthur, A.K. Kalvala, S.K. Surapaneni, M.S. Singh, Applications of cannabinoids in neuropathic pain: an updated review, Critical Reviews™ in Therapeutic Drug Carrier Systems 41(1) (2024).
